# Nosological Strictures

**Published:** 1819-03

**Authors:** T. Parkinson


*For the London Medical and Physical Journal.*

### *Nosological Strictures;* by T. Parkinson, M.D.

I BEG leave, through your Journal, to make some practical observations on a paper by Nathaniel Rumsey, giving an account of a "singular rheumatic affection, of an intermittent type."—Vide the *Edinburgh Medical and Surgical Journal* for July, 1818, page 342. I, at the same time, solicit the attention of your readers to a comparison of inflammatory affections in general with the case of Mr. Rumsey, who observes—"Several years ago, after recovering from a sore throat, which got well without producing ulceration or slough, I became ill from exposure to cold. Fever came on in the afternoon, with a violent pain of the abdomen, a great sense of distention and actual enlargement, with great flatulence. My own sensation was, that nothing would give relief but evacuations. An enema was given, with little or no good effect; yet, in the course of a few hours, I fell asleep, and awoke in the morning almost well, not expecting any renewal of disorder. But on the next evening I found myself suffering again precisely in the same manner," and so on for three successive days; after which he began to suspect intermittent rheumatism of the abdominal muscles, took the bark, and escaped the paroxysm on the following day. He adds, "My speedy recovery convinced me that the attack was intermittent rheumatism, not inflammatory, as might with reason have been suspected." Again he says, "I thought it a remarkable fact, not aware that rheumatism affected the muscles of the abdomen in this way; and still more remarkable that, by their vicinity to the bowels, without any intelligible or direct communication, the viscera should be also affected, as the flatus and distention proved."

His own case is followed by one similar in symptoms and situation; succeeded by another of the same character, intermittent, but situated at first in the face and teeth, but ended in the abdominal muscles. The bark was the efficient remedy.

The observations I wish to make, I shall limit to such as have been deduced from practical facts.

1. Rheumatismus is a term intended to denote inflammation of the moving solid, called muscle; and all muscles, be their form or situation whatever they may, are the subjects of it, not excepting the heart.

2. Inflammation of the moving solid; as well as inflammation of any other solid, attacks with different degrees of violence; but, in almost all cases of rheumatismus, evident remissions are discoverable, and that even in the beginning and early progress of the disease; and it is further to be observed, that, when any individual muscle is attacked, the first symptom is acute pain of that muscle, much increased by its own action, which in many instances cannot be suspended, or even controled, by the will. Such pain is succeeded by a sense of coldness of the extremities, rigor, followed by increased heat, thirst, a quick and full pulse, and highly-coloured urine. After some hours, either a partial diaphoresis, or a general perspiration, anticipates a considerable remission of pain, and a concomitant diminution of the constitutional affection commonly called fever. After such remission, which may be of longer or shorter duration, the same muscle, or perhaps more commonly another muscle, is attacked as above described, followed by all the *consectaria pyrexia* of Cullen, &c. After some hours, a remission both of the local pain and the constitutional sufferings are remarkable; and again, another accession of inflammation, with all its consectaria: the disease thus alternating between a greater and a less degree of intensity.

In this manner the disease proceeds, until it wears itself out, or is removed by medical treatment; and, under either circumstance, it most commonly, perhaps always, after an uncertain period, assumes a new type—intermittent; that is, the intervals are of determinate durations, and totally free from pain and fever, though the paroxysms are more or less severe, the urine depositing a lateritious sediment.

It must be remembered, that the solid denominated muscle is not limited to that portion which is the acting or contracting portion: it comprehends, also, all its modifications, the tendinous portion, the periosteum and the fascial portion; it moreover appears that these modifications of muscle, especially the fascial and periosteal, are more acutely painful under inflammation than the body of the muscle itself.

Let it be observed, that this character of inflammation is not peculiar to the muscle; it is common to all solids of the first order, as will be fully illustrated in my "Synopsis Zoo-Nosologiæ."

This order is my first, which I have denominated PLATYMORPHIA, from πλατυς and μορφοω. It comprehends all the solids in the animal system, excepting adipose substance and glands; and, on a careful retrospect of practical observations, but more especially from future enquiries, it will, I doubt not, be acknowledged that inflammation in all of them is characterized by the same local and general symptoms, is governed by the same laws in its progress, and in its fatal termination; viz.—the local inflammation is Erythema, and the constitutional affection Typhoid: consequently, the fatal termination is by gangrene and typhus.

As the first order of solids is Platymorphia, so the order of inflammation is designated by the qualifying adjective DIFFUSUS.

Inflammation is objected to as a classical term; consequently the whole family,—such as Pyrexia, Phlogosis, Phlegmone, &c.—will be rejected in my Synopsis, and HYPERBIOSIS will be substituted for Pyrexia, as a classical term. Hence we have Hyperbiosis diffusa for the class and order for inflammation in the first order of solids.

The second order of solids I have denominated SPHÆRIMORPHIA, from Σφαιρα μορφοω.

This order embraces adipose substance and glands only; and, in strict conformity, I have denominated inflammation in this order Hyperbiosis Spherica. Observe, that, in its progress and in its fatal termination, it is governed by the same inflexible laws; viz.—assumes a circumscribable tumor, and terminates in abscess; or otherwise, it resolves into another series, as Phylakesis or Symphylakesis.

The trifling difference in the symptoms of inflammation of the first order of solids, or Hyperbiosis diffusa, depends chiefly, perhaps altogether, upon the anatomical construction of the solid which is diseased; and it is worthy of remark, that inflammation of one solid is frequently mistaken for that of another, and consequently obtains a denomination at variance with the true nature of the disease: Pleurodyne for inflammation of the intercostal muscles, &c. for instance: and, let me add, this affection is of the same identical character as inflammation of the abdominal muscles represented by intermittent rheumatism in Mr. Rumsey's case. So long, however, as inflammation of muscles is denoted by the term rheumatism, what is called Pleurodyne should be denominated Rheumatismus intercostalis.

But it is of the highest importance to give to diseases suitable and appropriate appellations, and such appellations should be strictly in accordance with a settled nosological nomenclature, of chaste derivation and determinate import; and, until such a nomenclature is established and adhered to, it will be impossible to free medical science from that opprobrious jargon which has so long infested it.

---

*For the London Medical and Physical Journal.*

